# Manganese molybdate nanoflakes on silicon microchannel plates as novel nano energetic material

**DOI:** 10.1098/rsos.171229

**Published:** 2017-12-06

**Authors:** Chi Zhang, Dajun Wu, Liming Shi, Yiping Zhu, Dayuan Xiong, Shaohui Xu, Rong Huang, Ruijuan Qi, Wenchao Zhang, Lianwei Wang, Paul K. Chu

**Affiliations:** 1 Key Laboratory of Polar Materials and Devices, Ministry of Education, and Department of Electronic Engineering, East China Normal University, Shanghai 200241, People's Republic of China; 2 Shanghai Key Laboratory of Multidimensional Information Processing, East China Normal University, Shanghai 200241, People's Republic of China; 3 School of Physics and Electronic Engineering, Changshu Institute of Technology, Suzhou 215500, People's Republic of China; 4 School of Chemical Engineering, Nanjing University of Science and Technology, Nanjing 210094, People's Republic of China; 5 Department of Physics and Materials Science, City University of Hong Kong, Tat Chee Avenue, Kowloon, Hong Kong, People's Republic of China

**Keywords:** MnMoO_4_, nanoflakes, Si-MCP, thermite, energetic materials

## Abstract

Nano energetic materials have attracted great attention recently owing to their potential applications for both civilian and military purposes. By introducing silicon microchannel plates (Si-MCPs) three-dimensional (3D)-ordered structures, monocrystalline MnMoO_4_ with a size of tens of micrometres and polycrystalline MnMoO_4_ nanoflakes are produced on the surface and sidewall of nickel-coated Si-MCP, respectively. The MnMoO_4_ crystals ripen controllably forming polycrystalline nanoflakes with lattice fringes of 0.542 nm corresponding to the $\left(\bar{1}11\right)$ plane on the sidewall. And these MnMoO_4_ nanoflakes show apparent thermite performance which is rarely reported and represents MnMoO_4_ becoming a new category of energetic materials after nanocrystallization. Additionally, the nanocrystallization mechanism is interpreted by ionic diffusion caused by 3D structure. The results indicate that the Si-MCP is a promising substrate for nanocrystallization of energetic materials such as MnMoO_4_.

## 1. Introduction

Energetic materials (explosives, propellants and thermites) have attracted more and more attention recently due to wide applications in both civilian and military. Not only traditional explosives but also the variety and number of materials with good thermal stability and insensitivity have been required intensely. In the past, the traditional crystallite materials played the primary roles, such as amino/aminonitro heterocycles [1], azides [2] and polynitrogen compounds [3,4]. With the rapid development of nanoscience and nanotechnology, morphology is one of the important factors for the sensitivity of energetic materials [5]. In particular, nano energetic materials show dramatic improvement of the energy release, the reliability in initiation, detonation velocity and charge density. In addition, it also causes the decrease in the explosion critical radius [6,7]. Nanocrystalline compounds are known as metastable intermolecular composites (MIC) in the class of thermite materials [8,9], which are pyrotechnic compositions of metal powder fuels and oxidizers. Aluminium is the most common choice for fuels because of its low cost and high boiling point. And oxidizers are generally metallic oxides, such as iron [10], molybdenum [11] and manganese oxides [12]. Combined with aluminium, these thermite materials have high combustion efficiency, fast energy-releasing rate and good safety performance. Sun *et al.* [13] composed aluminium and molybdenum trioxide nanoparticles which indicate that the reactivity of nanoparticles is significantly higher than that of micrometre-size samples with a reaction range of 200–300 kJ mol^−1^. Comet *et al.* [14] mixed MnO_2_ and Al nanoparticles in hexane solution as high-energy thermite. Granier & Pantoya [15] studied the ignition and combustion behaviours of nanocomposite Al/MoO_3_.

Recently, binary metal oxides have attracted much interest due to their novel chemical and physical properties. As we know, molybdenum metal has the ability to form stable oxides with a series of metals (Mg, Mn, Fe, Co, Ni, Zn, etc.). The reports on these molybdates reveal the extraordinary performance of binary metal oxides which represents an interesting group of properties [16,17]. Minakshi *et al.* [18] synthesized nanoscale ternary molybdate (Mn_0.33_Nio_0.33_Co_0.33_MoO_4_) using biopolymer as a precursor to improve the energy storage performance. Currently, since both manganate-oxides and molybdate-oxides materials are common MIC materials, nanocrystalline manganese molybdate is proposed in an application as an energetic material. Combined with Al as fuel, nanocrystalline MnMoO_4_ is the oxidizer of thermite materials. MnMoO_4_ has the wolframite structure with the smaller bivalent manganese cations and molybdenum atoms having an overall sixfold coordination [19]. Manganese molybdate obtained by previous methods (such as sol–gel method, a solid-state reaction at high temperature and hydrothermal process) was often crystallized with crystal size larger than few tens of micrometres rather than nanocrystalline materials [20,21]. Lei *et al.* [22] prepared manganese molybdate rods and hollow olive-like spheres in micrometre-scale. Senthilkumar *et al*. [21] reported MnMoO_4_ with the size of about 20 µm. Watcharatharapong *et al.* [23] prepared manganese molybdate rods with a length of 10 µm. These MnMoO_4_ obtained are large crystallite materials with micrometre dimension. Nevertheless, Mu *et al.* [24] reported ultrathin manganese molybdate nanosheets of about 200 nm grown on Ni foam. Cao *et al.* [25] reported MnMoO_4_ nanoplates grown on a Ni foam substrate with the size of 500 nm. It was found that MnMoO_4_ grown on three-dimensional (3D) substrate is preferred to be nanocrystalline structure. However, the specific mechanism was barely studied. Hence in this study, a patterned 3D substrate, silicon microchannel plate (Si-MCP), is proposed to act as a substrate due to larger aspect ratio and better orderliness than Ni foam. Si-MCP is arranged with large aspect ratio lattices with length of 5 µm and depth of 300 µm. Manganese molybdate polycrystalline nanoflakes are synthesized on this ordered substrate. The growth mechanism is analysed and discussed more easily. Then, MnMoO_4_ polycrystalline nanoflakes exhibit novel properties of energetic materials. To research the crystal growth mechanism, a plane substrate is adopted as comparison. Otherwise, different concentrations of solution are prepared to contribute to analysing the growth process of the nanoflakes. Last but not least, the nanocrystallization mechanism based on 3D substrate is expounded in detail.

## 2. Material and methods

### 2.1. Synthesis and crystal growth

Analytical grade chemical reagents were used without further purification. Manganese chloride (MnCl_2_), ammonium molybdate tetrahydrate ((NH_4_)_6_Mo_7_O_24_·4H_2_O) and other reagents were purchased from Sinopharm Chemical Reagent Co. Ltd. The de-ionized (DI) water used to prepare the solutions had a resistivity of 18 MΩ cm.

The Si-MCP was fabricated by electrochemical etching and details about the preparation procedures are available in Yuan *et al*. [26]. A nickel film was deposited on the surface and inner wall of the Si-MCP by flow deposition to produce the MECN (macro and electrically conductive network) in which the Ni film plays the role of improving the adhesion and electrical properties [27].

Then, 1 M (NH_4_)_6_Mo_7_O_24 _· 4H_2_O and 7 M MnCl_2_ solutions were prepared with DI water at room temperature. The (NH_4_)_6_Mo_7_O_24_ solution was added slowly to the MnCl_2_ solution under stirring to form a homogeneous solution and the pH was adjusted to about 7 by an NH_3_ solution [28]. After the precursor solution was transferred to a Teflon-lined stainless steel autoclave liner, the Ni/Si-MCP was added into the solution after immersing in 0.1% Triton X-100 solution for 30 s. The autoclave was then sealed and heated to 140°C for 15 min to produce a deep-brown precipitate containing MnMoO_4_ on the surface of the Ni-coated Si-MCP. After cooling to room temperature in air, the product was taken out, rinsed ultrasonically with DI water and ethanol sequentially three times and vacuum dried at 80°C for 6 h. An aluminium film was deposited on the MnMoO_4_/Ni/Si-MCP as fuel by electroplating which is reported in Shi *et al*. [29].

### 2.2. Sample characterization

X-ray diffraction (XRD) was performed with a Rigaku RINT2000. The surface morphology and microstructure of the MECN and MnMoO_4_ were examined using field-emission scanning electron microscopy (FE-SEM; JEOL, JSM-7001F, Japan) equipped with an energy-dispersive X-ray spectrometer. The Raman spectra were recorded from 200 to 1000 cm^−1^ on an Olympus BX41 Raman Microprobe using a 524.4 nm argon ion laser. Transmission electron microscopy (TEM) was conducted with a JEOL JEM-2100 FEF. The onset temperature and energy release were monitored by differential scanning calorimetry (DSC) and thermogravimetric analysis (TGA) (Mettler Toledo, TGA/DSC 1).

## 3. Results and discussion

### 3.1. Characterization of the hierarchical MnMoO_4_/Ni/Si-MCP

**Figure 1.**
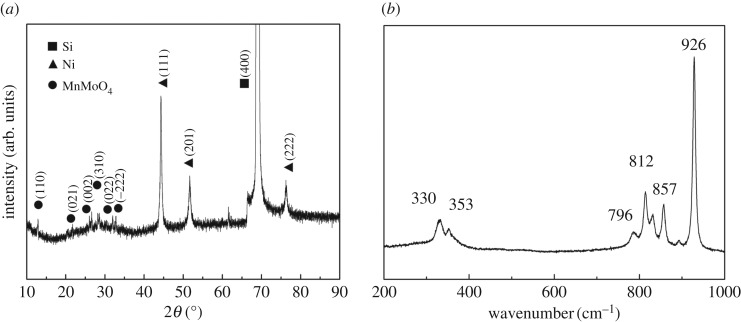
(*a*) XRD pattern of the MnMoO_4_/Ni/Si-MCP and (*b*) Raman spectrum of the synthesized MnMoO_4_/Ni/Si-MCP.

To analyse the phase structure of the sample, XRD measurement is conducted. The XRD pattern in figure 1*a* (MnMoO_4_: JCPD card 72-0285) indicates the formation of manganese molybdate with a monoclinic crystal system with the C2/m (12) space group. The obtained diffraction peaks at 12°, 22°, 26°, 28°, 32° and 33° are indexed to the corresponding crystallographic planes of (110), (021), (002), (310), (022) and (−222), respectively. At the same time, the pattern also presents three Ni peaks: (111) at 44°, (201) at 52° and (222) at 76°; and one strong Si peak: (400) at 69° (Ni: JCPD card 70-0989 and Si: JCPD card77-2109). The structure of MnMoO_4_ was further measured by Raman spectroscopy. Figure 1*b* reveals a high-intensity line at 926 cm^−1^, medium-intensity lines at 812 and 857 cm^−1^ and low-intensity lines at 330 cm^−1^, 353 cm^−1^ and 796 cm^−1^, which are the characteristic bands of MnMoO_4_ [30]. The highest intensity band corresponds to the Mo(1)O(2) symmetric stretching vibration [31].

**Figure 2.**
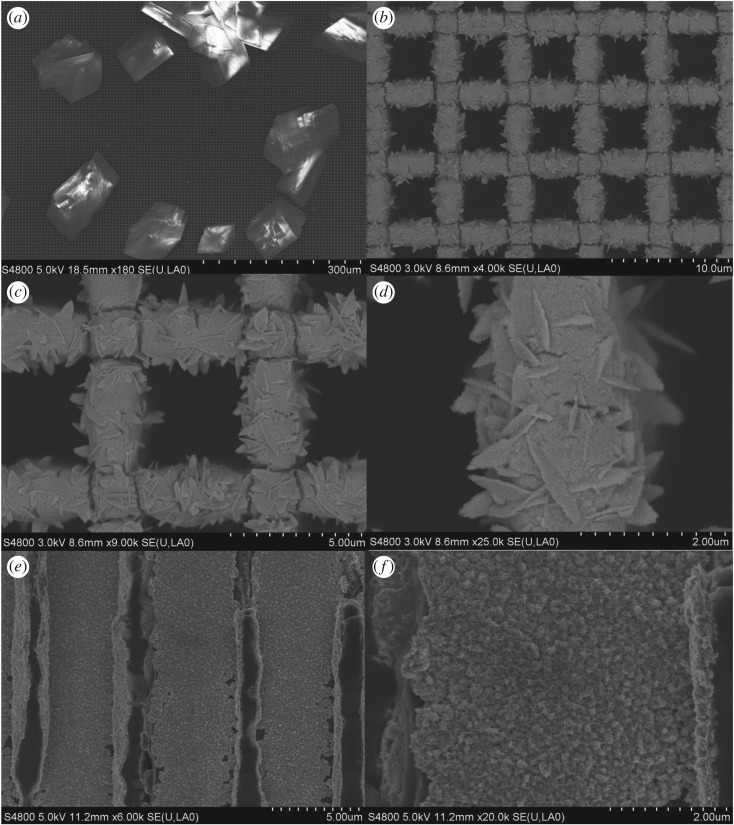
(*a*) FE-SEM image of top surface of MnMoO_4_; (*b–d*) magnified images of that in (*a*); (*e*) FE-SEM image of the cross section of MnMoO_4_; (*f*) magnification image of that in (*e*).

Figure 2*a–f* displays the FE-SEM images of the MnMoO_4_/Ni/Si-MCP. Figure 2*a* reveals large crystallite MnMoO_4_ with micro size on the surface of the Ni/Si-MCP similar to previous results [28]. The higher magnification FE-SEM images in figure 2*b–d* show that the MnMoO_4_ on the microchannel has the shape of a half moon or arched willow leaves. The nanoflakes on the Ni layer are interconnected with each other forming an ordered array with an open network. The MnMoO_4_ nanoflakes on the inner side wall of the Ni/Si-MCP shown in figure 2*e,f* disclose smaller and denser nanoflakes. Three different morphologies of MnMoO_4_ are present on the microchannel at the same time. And they can be classified into two kinds of materials: large crystallite MnMoO_4_ and MnMoO_4_ nanoflakes.

**Figure 3.**
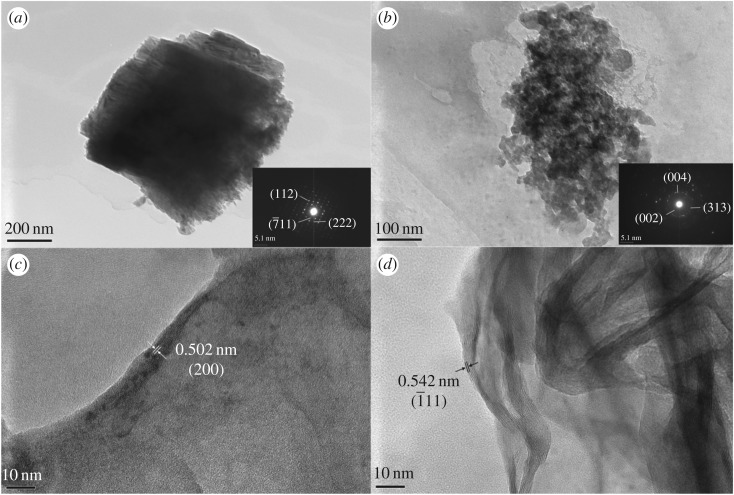
(*a*) TEM image of crystallite of MnMoO_4_; (*b*) TEM image of MnMoO_4_ nanoflakes; (*c*) magnified image of that in (*a*); (*d*) magnified image of that in (*b*).

To further analyse the difference between the large crystallites on the surface and nanoflakes on the microchannels, MnMoO4/Ni/Si-MCP is measured by TEM. Two different materials are separated into two samples. The sample with the crystallites shown in figure 3*a* is prepared by ultrasonication in ethanol for a sufficiently long time to make sure the crystallites are detached from the surface. And the nanoflakes sample shown in figure 3*b* is separated centrifugally from the fragments after etching in KOH solution. Figure 3*a,c* shows that the crystallites are monocrystals with an interplanar spacing of 0.502 nm corresponding to the (200) plane of MnMoO_4_. The selected-area electron diffraction pattern in figure 3*a* confirms the monocrystals as diffraction spots can be clearly observed [32]. Figure 3*b,d* depicts the TEM images of the nanoflakes. The nanoflakes are polycrystalline with a spacing of 0.542 nm corresponding to the $\left(\bar{1}11\right)$ plane. Figure 3*b* reveals the polycrystalline nature of the nanoflakes.

**Figure 4.**
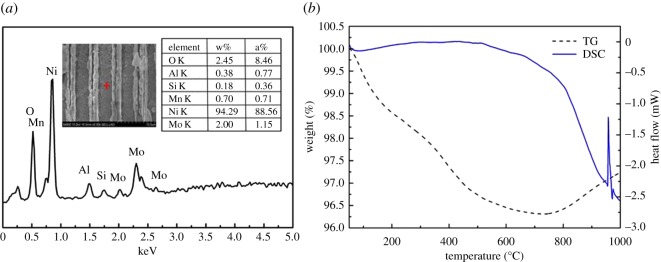
(*a*) EDS spectrum of Al/MnMoO_4_/Ni/Si-MCP. (*b*) DSC and TGA curves acquired from Al/MnMoO_4_/Ni/Si-MCP.

After coating aluminium film, the composite sample is verified by energy-dispersive X-ray spectroscopy (EDS) as shown in figure 4*a*. According to the inset SEM image Al film is deposited on the MnMoO_4_/Ni/Si-MCP. By comparison with figure 2*c*, the Al film is deposited uniformly on the MnMoO_4_ nanoflakes without destroying the morphology of MnMoO_4_. The elements at the position marked by the red cross are analysed as shown in the table. It discloses the presence of Mn, Mo, O, Ni, Si and especially Al. To study the performance of the sample as energetic material, DSC/TGA experiments are conducted at a temperature from 50°C to 1100°C with a heating rate of 10°C min^−1^ in 50 sccm N_2_. The thermal analysis results are presented in figure 4*b* based on the mass-corrected values. As the temperature rises, weight loss occurs gradually and at 730°C, the weight curve rises because of the thermite reaction between Al and oxygen/vapour in the sample and environment. The energy generated by the thermite reaction is quite obvious at 960°C. The peak in the heat flow curve reveals the activation energy of the thermite reaction [33].The prominent thermite performance in the DSC/TGA analyses reveals that the nanocrystallization improves the properties of MnMoO_4_ and makes it to be a new kind of energetic material successfully and meaningfully.

### 3.2. Nanocrystallization mechanism

When the (NH_4_)_6_Mo_7_O_24_ solution is mixed with the MnCl_2_ solution, a supersaturated solution is obtained and tiny crystalline nuclei are formed initially. As a result of nucleation regularity, these nuclei are transferred to both the surface and inner wall of the Si-MCP evenly. In the supersaturated solution, the particles connect the crystals formed afterwards. Following hydrothermal crystallization and ripening, the larger particles grow at the expense of smaller ones in accordance with the Gibbs–Thomson law [34–37]. Formation of MnMoO_4_ on the surface has a similar mechanism.

**Figure 5.**
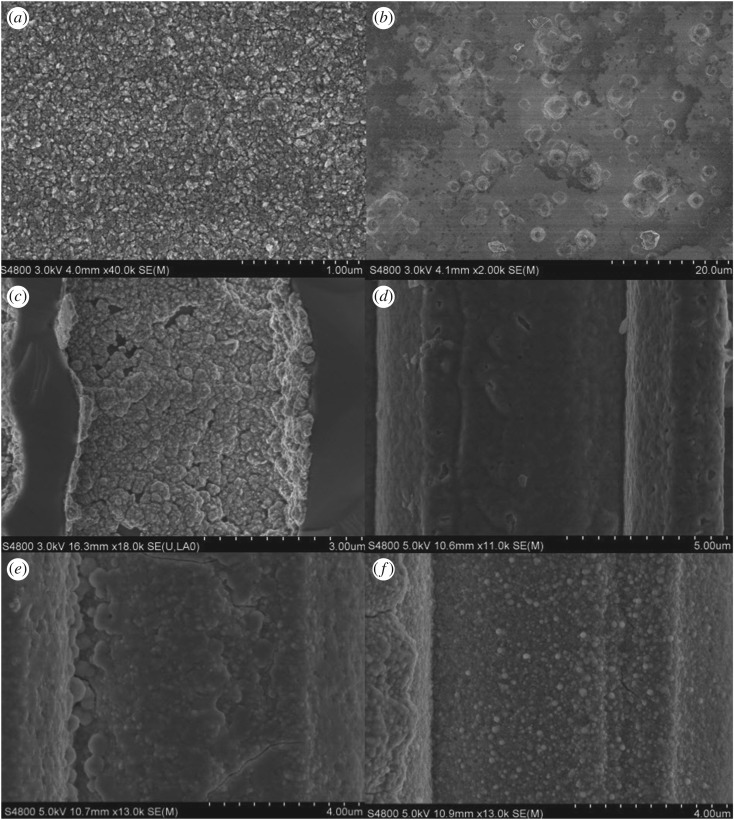
(*a*) FE-SEM image of the Ni/Si plane; (*b*) FE-SEM image of the MnMoO_4_/Ni/Si plane; (*c*) FE-SEM image of Ni/Si-MCP; (*d*) FE-SEM image of MnMoO_4_/Ni/Si-MCP (25% concentration); (*e*) FE-SEM image of MnMoO_4_/Ni/Si-MCP (50% concentration); (*f*) FE-SEM image of MnMoO_4_/Ni/Si-MCP (75% concentration).

Our results show that there are two different types of crystals on the MCP. To further assess the crystal growth mechanism, a sample fabricated on a flat silicon substrate is compared. As shown in figure 5*a*, the silicon substrate undergoes the electroless process similar to Si-MCP (figure 5*c*). Tiny nickel particles are deposited on the silicon substrate uniformly and the morphology is shown in figure 5*b*. There are no nano-structured crystals on the plane substrate but instead large MnMoO_4_ particles are observed, indicating that the MCP with a large aspect ratio is crucial to the formation of MnMoO_4_ nanoflakes. Different solution concentrations are also prepared. The samples immersed in solutions with concentrations 25%, 50% and 75% of the standard one are processed hydrothermally. As the concentration decreases, there are fewer particles on the sidewall as shown in figure 5*d–f*. When the concentration is 25% of the standard one, the deposited product is a thin film in lieu of particles, indicating that the concentration is also critical to the formation of the nano-structure. The small particles shown in figure 5*d* do not morph into large crystallites demonstrating that the structure of the Si-MCP affects the crystal growth.

**Figure 6.**
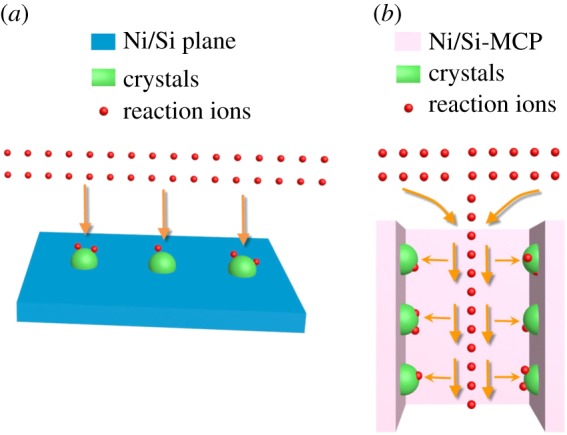
(*a*) Growth mechanism on planar Si and (*b*) growth mechanism on Si-MCP.

The crystal growth mechanism is illustrated in figure 6*a*. When the substrate is planar, the ions arrive at the surface from the solution evenly resulting in continuous and stable absorption and reaction. The particles grow to form large crystallites according to Gibbs–Thomson law. If the time is unlimited, the crystals grow until they reach the boundaries meaning that there is no effective absorption point. In comparison, on the channel plate with a large aspect ratio, the ions in the channel decrease as a result of the reaction in crystal ripening, as shown in figure 6*b*. The outer ions diffuse into the channels continuously due to the concentration gradient. The ions flow parallel to the sidewall on the Si-MCP and the flow direction is along the shearing direction of the crystals on the sidewall. The particles are separated by the shearing stress. The particles absorb and react on the entire surface evenly rather than preferentially according to the growth orientation [38–41]. Hence, the crystals cannot capture each other to form large crystallites thus forming the polycrystalline nanoflakes. The nanoflakes on the orifice of the microchannel grow by the same mechanism that ion flow obstructs the normal crystal growth. Since the ion flow on the orifice is not absolutely parallel to the side wall inside the channel, the nanoflakes are non-uniform.

## 4. Conclusion

A novel category of nano energetic material is designed and prepared with Al as fuel and MnMoO_4_ nanoflakes as oxidizer. By introducing the 3D microchannel structure, monocrystalline MnMoO_4_ with a size of tens of micrometres and polycrystalline nanoflakes are produced on the surface and sidewall of the nickel-coated Si-MCP, respectively. The MnMoO_4_ crystals have good crystallinity and high purity. The lattice fringes of large crystallite are 0.502 nm, corresponding to the (200) plane of MnMoO_4_. The MnMoO_4_ crystals ripen controllably forming polycrystalline nanoflakes with the lattice fringes of 0.542 nm corresponding to the $\left(\bar{1}11\right)$ plane on the sidewall. The synthesized MnMoO_4_ nanoflakes display unprecedented thermite performance which reveals the possibility of new application as energetic material. Last but not least, to research the growth mechanism, a flat silicon substrate was adopted as comparison. Samples with various concentrations of ions were also prepared as comparisons, and can be explained by ionic diffusion caused by 3D structure. The results indicate that the Si-MCP is a promising substrate for nanocrystallization of energetic materials such as MnMoO_4_.

## Data accessibility

http://dx.doi.org/10.5061/dryad.g1693 [42].
